# Preparation, Purification and Characterization of Antibacterial and ACE Inhibitory Peptides from Head Protein Hydrolysate of Kuruma Shrimp, *Marsupenaeus japonicus*

**DOI:** 10.3390/molecules28020894

**Published:** 2023-01-16

**Authors:** Jie Zhou, Qiuyu Han, Tomoyuki Koyama, Shoichiro Ishizaki

**Affiliations:** 1 Graduate School of Marine Science and Technology, Tokyo University of Marine Science and Technology, 4-5-7 Konan, Minato, Tokyo 108-8477, Japan; 2 College of Food Science and Technology, Shanghai Ocean University, Shanghai 201306, China

**Keywords:** kuruma shrimp, protein hydrolysate, antibacterial peptide, ACE inhibitory peptide

## Abstract

Kuruma shrimp *(Marsupenaeus japonicus*) heads, as the main by-product of the seafood processing industry, are rich in underutilized high-quality protein. After papain hydrolysis at 50 °C for 4 h, the protein hydrolysate of shrimp heads was found to show notable antibacterial and angiotensin I-converting enzyme (ACE) inhibitory activities. After purification using two stages of revered-phase high-performance liquid chromatography (RP-HPLC), the antibacterial peptide VTVP and the ACE inhibitory peptide ARL/I were successfully identified from most active fractions by LC–MS/MS. Peptide VTVP was a desirable hydrophobic peptide, with a MIC value in the range from 1.62 to 8.03 mM against all tested pathogens. Peptide ARL/I exhibited potent ACE inhibitory activity, with an IC50 value of 125.58 µM, and was found to be a competitive inhibitor based on the Lineweaver–Burk plot. Moreover, the result of the molecular docking simulation indicated that the interaction binding between ARL/I and ACE was mainly stabilized by hydrogen bonds, as well as forming a coordinate bond with the Zn^2+^ site. The purified peptides did not show hemolytic activity toward rabbit erythrocytes. To sum up, the bioactive peptides isolated from shrimp heads could be applicable for food or pharmaceutical areas as promising ingredients.

## 1. Introduction

Shrimp, which is an important member of the crustacean family, constitutes a major part of crustacean consumption in recent years [1]. While most shrimps are usually processed as meat due to the export demand and consumer’s preference, a large number of by-products such as head, shell, and tail portions has been generated in the processing process [2]. Shrimp heads, which account for approximately 30–50% of whole shrimp weight among different species, are rich in high-quality protein (50–65%, dry weight basis) [3], which is an excellent route to process the marine bioactive peptides with considerable pharmacological potential. The bioactive peptides, usually containing 2–20 amino acid residues in length, are encrypted within the sequence of parent protein and can be released during hydrolysis and/or food processing [4]. Since the 1990s, various kinds of bioactive peptides derived from marine organisms have been isolated and reported by numerous researchers [5]. Antibacterial and angiotensin I-converting enzyme (ACE) inhibitory peptides are ranked as the most widely studied bioactive peptides [6].

The widespread use of antibiotics has greatly facilitated the treatment of bacterial infections. Starting from the 1980s, however, the number of newfound antibiotic chemicals declined dramatically, and then a rapid increase in antibiotic-resistant bacteria was caused due to the abuse of traditional antibiotics [7,8]. Therefore, there is an urgent need for alternatives to traditional antibiotic chemotherapy. Antibacterial peptides are small molecules, composed of less than fifty amino acid residues. These active peptides are essential components of the innate immune system and produced by nearly all living organisms to defend against the invading microorganisms [7]. Accordingly, the research and analysis of antibacterial peptides applied to alternative antibacterial strategies have increased with each passing day. Several antibacterial peptides derived from crustaceans have been isolated and reported. Yang et al. [9] have predicted twenty potential antibacterial peptides from the hemocyanin of shrimp *Litopenaeus vannamei* and then showed that the peptide L1 (VNFLLHKIYGNIRYS) consisting of an α-helix and a β-turns structure possessed the highest antibacterial activity against test microorganisms. Dromidin, a novel antibacterial peptide (513.0 Da), was also isolated and identified from the hemolymph of the crab *Dromia dehaani* [10]. In addition, the potential antibacterial activity of enzymatic hydrolysates from crustaceans has been recently researched. Robert et al. [11] have demonstrated that the white shrimp (*L. vannamei*) head hydrolysate possesses a high content of low-molecular-weight peptides and showed antibacterial activity against different bacterial strains. Moreover, it has also been proved that the enzymatic hydrolysate of Atlantic rock crab (*Cancer irroratus*) by-products showed inhibitory activity against several Gram-negative and Gram-positive bacteria [12].

Hypertension, among the diseases with a global burden, is an important risk factor for cardiovascular disease (CVD) and is a major health problem that affects approximately 29% of adult population worldwide, which is expected to be 2.5 billion in the year 2024 [13]. ACE is a dipeptide hydrolase that plays a crucial physiological role in regulating blood pressure in vivo. ACE works mainly through the renin–angiotensin system (RAS) that hydrolyzes the inactive angiotensin-I converted to the potent vasoconstrictor angiotensin-II through cleaving the C-terminal His–Leu dipeptide by ACE action, resulting in peripheral vascular resistance and blood pressure elevation [14]. Furthermore, ACE can also hydrolyze bradykinin which has vasodilatation properties. Therefore, the use of ACE inhibitors is believed to lower hypertension and further prevent CVDs. Some synthetic drugs show a significant effect in treating hypertension and related diseases. However, some adverse side effects, such as cough, allergic reactions, taste disturbance, and skin rashes, have also inevitably been caused [15,16]. Thus, searching for a kind of ACE inhibitor without undesirable side effects has become a focal subject of public interest. In recent decades, various ACE inhibitory peptides derived from marine invertebrate sources have been reported extensively, including shrimp [17,18], clam [19], and oyster [20]. These findings suggest that the proteins of marine invertebrates may be a good source to produce ACE inhibitory peptides. These peptides have the potential to be used as effective alternatives for application in the pharmaceutical and health industries because of the increasing interest in the safety and economical cost of drugs.

Kuruma shrimp, *Marsupenaeus japonicus*, is among economically important species of shrimp culture worldwide. From the statistical information of the Food and Agriculture organization (FAO, www.fao.org/fishery/statistics/en, accessed on 1 November 2022), the global production of kuruma shrimp was approximately 58,000 tons in 2018. A large amount of high-quality protein, stored in shrimp by-products, provides sufficient raw material for research. Although several bioactive peptides have been isolated and characterized from various marine organism sources, only a few peptides have been reported from crustaceans. In particular, to our present knowledge, there are no studies which focused on the antibacterial and ACE inhibitory activities of protein hydrolysate from kuruma shrimp by-products both in vitro and in vivo. Therefore, the aim of this work was to investigate the antibacterial and ACE inhibitory activities of peptides derived from the head wastes of kuruma shrimp by papain hydrolysis.

## 2. Results and Discussions

### 2.1. Biochemical Characteristics of Protein Hydrolysate of Kuruma Shrimp Heads (KSHPH)

It is well known that many parameters influence the biochemical activities of protein hydrolysate, including protease specificity, protein substrate, and hydrolysis conditions [6,21]. In this research, papain was used to produce the bioactive peptides from the head wastes of kuruma shrimp. Papain has broad specificity for catalyzing the hydrolysis of peptide bonds that release peptides with amino acids, such as leucine and glycine. In addition, papain is particularly suited for cutting amino acids bearing a large hydrophobic side chain at the P2 position [22,23]. First of all, the antibacterial activity of KSHPH was investigated by using four bacterial strains. As shown in Table 1, 40 mg of KSHPH (dry weight) showed an obvious inhibition zone against *Staphylococcus aureus* and *Micrococcus luteus*, but no inhibitory activity against *Escherichia coli* and *Shewanella putrefaciens*.

Furthermore, significant ACE inhibitory activity was exhibited in KSHPH, with an IC_50_ value of 1.90 ± 0.03 mg/mL (Table 2). It has been reported that the IC_50_ values of hydrolysates from shrimp, shark meat, and mackerel bone are relatively lower than those from marine protein hydrolysates [24]. Since it was found that the KSHPH had moderate antibacterial and ACE inhibitory activities, the subsequent purification experiments were crucial.

**Figure 1:**
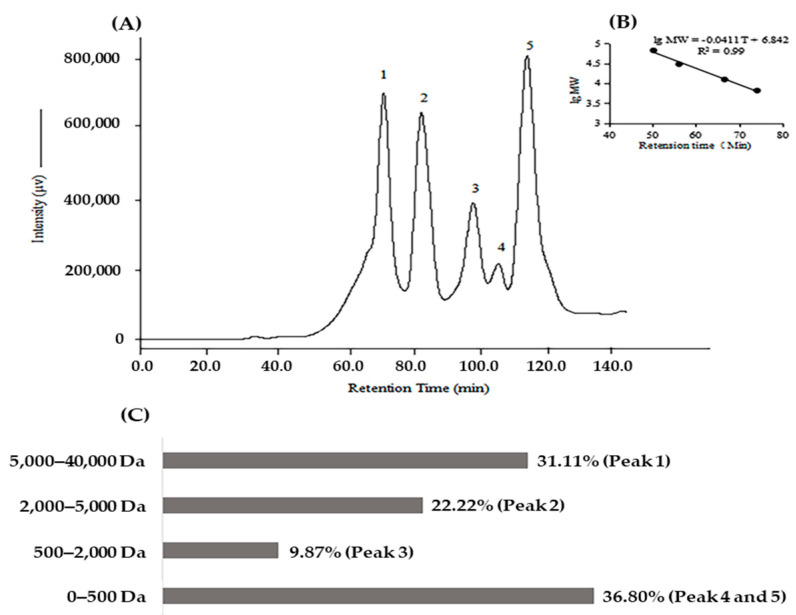
Size–exclusion chromatogram on a Superdex 75 10/300 GL column of KSHPH (**A**) and the calibration curve (**B**). Molecular weight distribution of KSHPH was calculated by calibration curve (**C**).

The molecular weight distribution was also analyzed by size-exclusion chromatography on a Superdex 75 10/300 GL column. The result was presented in Figure 1. Based on the calibration curve, the molecular weight of KSHPH was less than 40,000 Da. The protein hydrolysate had a high content of soluble peptides with a molecular weight less than 5000 Da (68.89%) and a considerable number of oligopeptides existed in it. This mass range indicated that the KSHPH had an appropriate degree of hydrolysis, which is important for generating bioactive peptides.

### 2.2. Purification of Antibacterial and ACE Inhibitory Peptides

**Figure 2:**
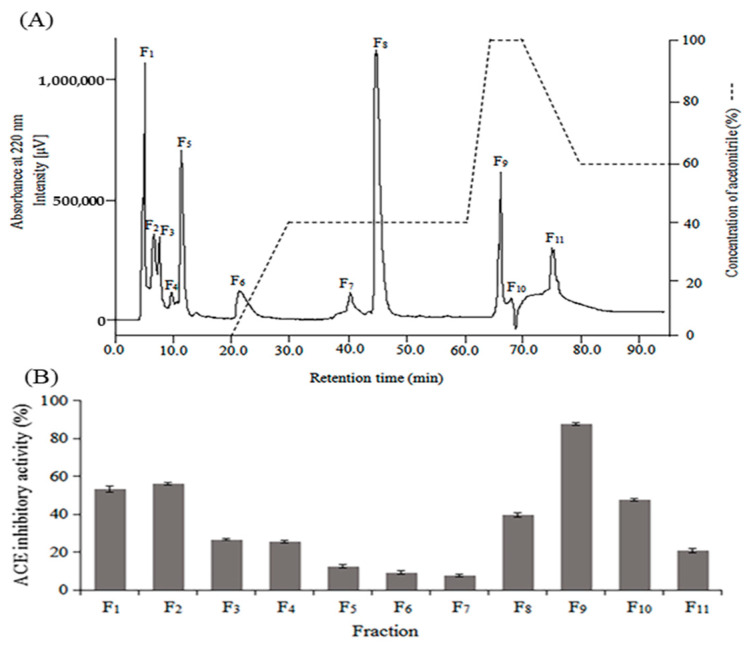
Purification profile of KSHPH separated by RP-HPLC using a TSKgel ODS-80TM column (**A**). Protein hydrolysate was eluted by a linear gradient of acetonitrile (0~20 min, 100% A; 20~30 min, 100% A→60% A/40% B; 30~60 min, 60% A/40% B; 60~65 min, 60% A/40% B→100% B; 65~70 min, 100% B; 70~80 min, 100% B→40% A/60% B) at a flow rate of 1.0 mL/min. ACE inhibitory activity of each fraction (F_1_–F_11_) was measured at a concentration of 8 mg/mL (**B**).

Enzymatic hydrolysis of kuruma shrimp head wastes generates a complex mixture of active and inactive peptides with different molecular weights and amino acid sequences. To purify the antibacterial and ACE inhibitory peptides, KSHPH was separated by two stages of RP-HPLC. The elution profile of KSHPH was monitored at a wavelength of 220 nm. As shown in Figure 2A, eleven major fractions (F_1_–F_11_) associated with each peak were collected separately, lyophilized, and then evaluated for antibacterial and ACE inhibitory activities.

Among these fractions, F_9_ exhibited the strongest antibacterial activity against all the test strains, with a wide range of MIC values from 5.00 to 16.67 mg/mL (Table 3).

**Figure 3:**
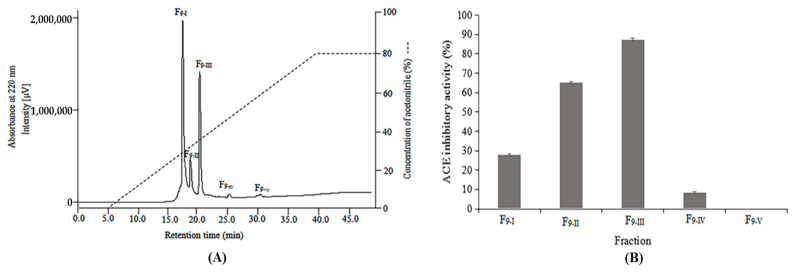
Purification profile of KSHPH-F_9_ separated by RP-HPLC using a Mightysil RP-18 column (**A**). Peptide fraction was eluted by a linear gradient of acetonitrile (0~5 min, 100% A; 5~40 min, 100% A→20% A/80% B; 40~50 min, 20% A/80% B.) at a flow rate of 0.5 mL/min. ACE inhibitory activity of each fraction (F_9-I_–F_9-V_) was measured at a concentration of 0.5 mg/mL (**B**).

ACE inhibitory activity was widely observed in all fractions at a concentration of 8 mg/mL, and the highest inhibitory activity (87.42%) was also shown in F_9_ (Figure 2B) with an IC_50_ value of 0.97 ± 0.04 mg/mL (Table 2). Hence, the F_9_ was subjected to a further purification stage on a Mightysil RP-18 column and fractionated into five major absorbance peaks (F_9-I_–F_9-V_) (Figure 3A). After antibacterial and ACE inhibitory activities were determined, the most potent antibacterial activity was found in F_9-I_, and the highest ACE inhibitory activity was observed in F_9-III_. The antibacterial efficacy of F_9-I_ was expressed as the MIC value, with a specific value between 0.67 and 3.33 mg/mL (Table 3). For the four bacterial strains, Gram-positive ones (*S. aureus* and *M. luteus*) were noted to show the higher rates of sensitivity to F_9-I_. After the second stage of RP-HPLC purification, the MIC value of F_9-I_ was approximately 5–7 fold more efficient than F_9_. In addition, as illuminated in Figure 3B, F_9-III_ displayed the highest ACE inhibitory activity (87.30%) at a test concentration of 0.5 mg/mL, with an IC_50_ value of 0.045 ± 0.005 mg/mL. As a results, F_9-III_ was purified approximately 42.22 fold from KSHPH using two stages of RP-HPLC purification.

### 2.3. Amino Acid Sequence of the Purified Peptide

**Figure 4:**
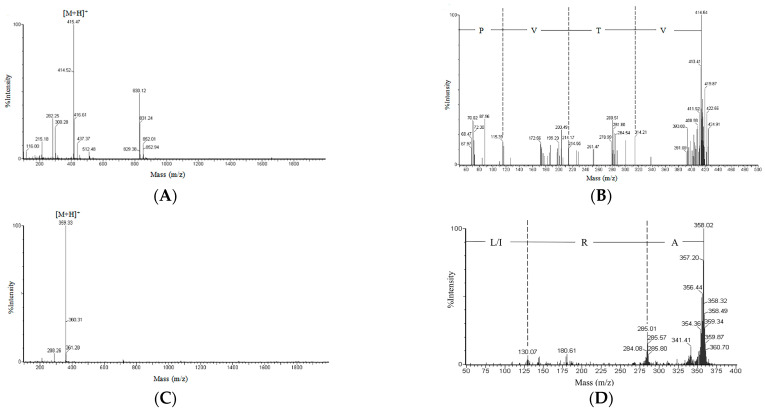
Identification of the molecular mass and amino acid sequence of active peptide fractions. ESI–MS spectrum of KSHPH-F_9-I_ (**A**), ESI–MS/MS spectrum of the precursor ion *m*/*z* 415.47 from MS of KSHPH-F_9-I_ (**B**), ESI–MS spectrum of KSHPH-F_9-III_ (**C**), and ESI–MS/MS spectrum of the precursor ion *m*/*z* 359.33 from MS of KSHPH-F_9-III_ (**D**).

The most potent active fractions (F_9-I_ and F_9-III_) were determined by LC–MS for molecular mass and LC–MS/MS for amino acid sequence. The peak purity of F_9-I_ and F_9-III_ was more than 98%. The low impurity content of fractions indicated that these purified fractions were suitable for amino acid sequence identification. The MS and MS/MS spectrum charts of target fractions were shown in Figure 4.

The accurate relative molecular mass of F_9-I_, deduced from the *m*/*z* value of [M + H]^+^ by subtraction of one mass unit for the attached proton, was 414.47 Da (Figure 4A). The amino acid sequence of F_9-I_ was identified to be Val−Thr−Val−Pro (VTVP) (Figure 4B). Similarly, it can be calculated that the precise relative molecular mass of F_9-III_ was 358.33 Da (Figure 4C), and the amino acid sequence was determined to be Ala−Arg−Leu/Ile (ARL/I) (Figure 4D).

The antibacterial peptide (VTVP) in the present study was a tetrapeptide and has no net charge in neutral solution. Retrieving the BIOPEP database (www.uwm.edu.pl/biochemia/index.php/en/biopep, accessed on 15 November 2022) and Antimicrobial Peptide Database (aps.unmc.edu/AP/main.php, accessed on 17 November 2022), it has high possibility that this is the first time the peptide VTVP with antibacterial activity was isolated from protein resources. The grand average of hydropathicity (GRAVY) value of the peptide VTVP was 1.53 > 0, and the hydrophobic ratio was 50%; these results indicated that this peptide shows a reasonable hydrophobic structure. Actually, numbers of antibacterial peptides have been determined to be short peptides (2–20 amino acids), of which nearly 50% are hydrophobic residues [25,26]. The hydrophobicity of antibacterial peptides showed an important effect to enable the peptide to penetrate bacterial cells and induce membrane lysis. An excessive increased ratio of hydrophobicity of the antibacterial peptide was correlated to its low selectivity and toxicity toward mammalian cells, and would hold the peptide back to transport to the target microorganism [27,28]. Thus, proper hydrophobicity of the antibacterial peptide is crucial. The 3D model and software analysis indicated that the secondary structure of VTVP is mainly composed of random coil.

Random coil is a desirable confirmation that can protect the peptide against adverse conditions in actual environments [29]. In addition, the peptide VTVP was too short to form a typical amphiphilic structure such as α-helical peptide. However, the alternating hydrophobic and hydrophilic amino acid sequence of VTVP may also generate a kind of amphiphilic structure that plays a significant role on peptide contact with the cell membrane of bacteria. The alternating tetrapeptide sequence has been reported for the preparation of nanomaterials [30]. As reported in Table 3, the short peptide VTVP exhibited the widest range of antibacterial activity with MIC values ranging between 1.62 and 8.03 mM. These results were highly in agreement with the previous findings reported by Bougherra et al. [31] and Kobbi et al. [26], which showed that the active peptide is a short peptide and does not have the classical characteristic of the antibacterial peptides.

The ACE inhibitory tri-peptides, ARL/I, have not been isolated from other resources. The highest ACE inhibitory potency of ARL/I may be associated with the low peptide size and the suitable hydrophobic ratio of ARL/I (66.67%). Peptide size and the hydrophobicity of the sequence are two critical indexes in determining the affinity with ACE active site. Since the ACE was a complex polymer protein with lots of α-helical structures, the binding channel of ACE is too narrow to accommodate the large peptides [32]. Furthermore, a highly hydrophilic property could make the peptide inaccessible to the active site of ACE, as the hydrophilic–hydrophobic balance is a vital factor in biologically active molecules [33]. Wu et al. [34] studied the quantitative structure–activity relationships of ACE inhibitory peptides composed of 168 dipeptides and 140 tripeptides based on a database. The results indicated that the most favorable tripeptides residues were the aromatic amino acids in the carboxyl terminus, while the positively charged amino acids and hydrophobic amino acids were preferred for the middle position and the amino terminus, respectively [34]. In the present study, the peptide ARL/I did have the Arg residue, a positively charged amino acid located in the middle position, and the Ala residue, a hydrophobic amino acid located in the amino terminus. The carboxyl terminal amino acid of the peptide ARL/I is leucine or isoleucine, which is a hydrophobic amino acid. Actually, the hydrophobic amino acid residues at the amino or carboxyl terminus were also essential to form the ACE inhibitory peptides [35,36]. Thus, the tripeptide (ARL/I) has potent ACE inhibitory activity with an IC_50_ value of 125.58 µM, which was calculated based on the molecular mass of ARL/I and Table 2, which may provide a drug candidate for treatment of hypertension.

### 2.4. ACE Inhibition Pattern of the ACE Inhibitory Peptide

**Figure 5:**
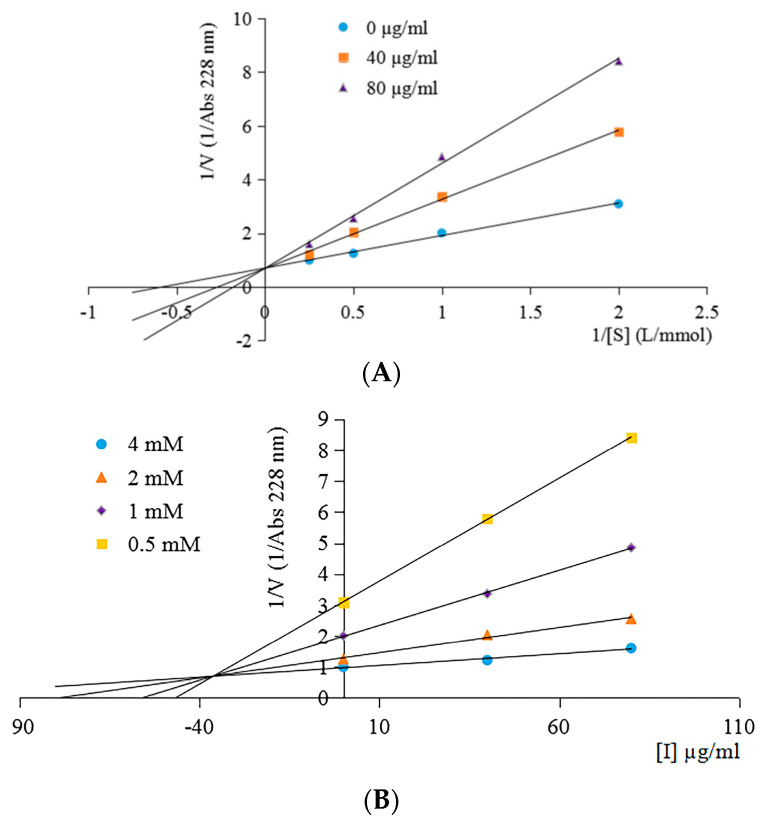
Lineweaver–Burk plot of ACE inhibition of the purified peptide (KSHPH−F_9-III_) at concentrations of 40 and 80 µg/mL (**A**); the Dixon plot was used to calculate the inhibition constant Ki of the peptide (**B**).

The ACE inhibition pattern of the purified peptide ARL/I explained the characteristics of peptide binding to ACE and inhibited the enzyme activity. As shown in Figure 5A, the line of varying concentrations of the purified peptide intersecting at common intercepts on y-axis indicated that the V_max_ (maximum velocity) value of the enzyme reaction remains unchanged. However, the K_m_ (Michaelis–Menten constant) values of the reaction increased with the rise in inhibitor concentration. These results indicate that the peptide was a competitive inhibitor, and the inhibitor constant Ki value was 36.19 ± 0.28 µg/mL (Figure 5B). The competitive inhibitor suggested that the inhibitor can enter the interior of ACE and interact with the ACE active sites and prevent substrate binding [37].

### 2.5. Molecular Mechanism between the ACE Inhibitory Peptide and ACE

**Figure 6:**
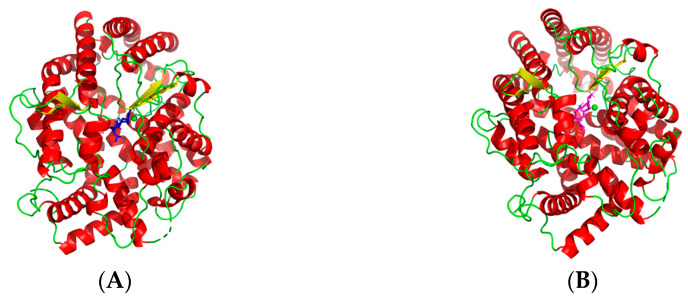

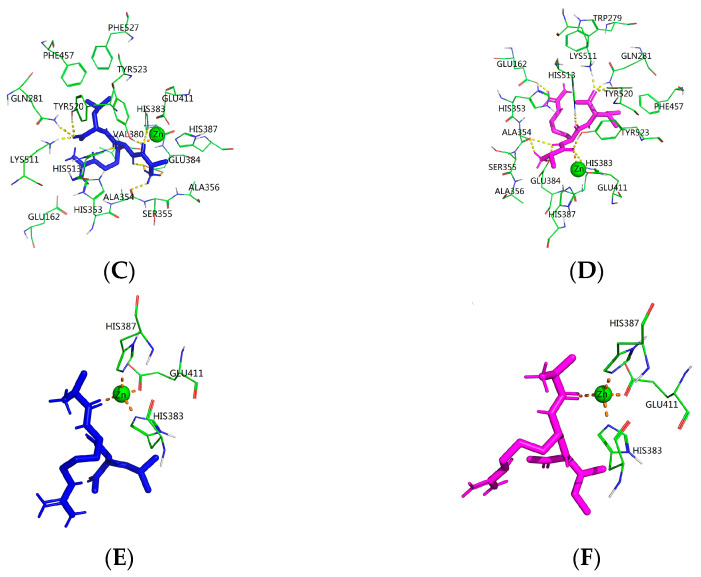
Molecular docking between ACE and ARL/I. General overviews of docking pose of ARL and ARI at the ACE site are shown in (**A**,**B**), respectively. The blue stick mode is ARL; the magenta stick mode is ARI. (**C**,**D**) mean details of the interaction between ACE and peptide ARL and ARI, respectively. The hydrogen bonds are shown in yellow dashed lines and zinc coordination bonds are in oranges dashed lines. Schematic views of ARL– and ARI–zinc coordination at the ACE active site are indicated in (**E**,**F**), respectively.

Despite the ACE inhibitory peptides as an alternative for control of hypertension have been extensively studied, the precise molecular mechanisms between active peptides and ACE catalytic site are not fully understood. Therefore, the molecular docking simulation between the purified peptide (ARL/I) and ACE was analyzed using the flexible docking tool of AutoDock 4.2 software. The docking study of the tripeptide ARL/I at the ACE catalytic sites in the presence of Zn(II) showed the best returned pose in Figure 6, with binding energy values of −9.22 kJ/mol (ARL) and −9.34 kJ/mol (ARI), respectively.

As exhibited in Figure 6A,B, the small ligand (ARL/I) successfully entered in the deep narrow channel of the ACE active site. Usually, the best pose of enzyme–inhibitor complex was stabilized by hydrogen bonds, and hydrophobic, hydrophilic and electrostatic interactions [38,39]. The hydrogen bond interaction force was particularly important for the docking complex among these forces [38]. Details of the peptide ARL/I interaction with ACE residues after docking are shown in Figure 6C,D—the peptide ARL formed ten hydrogen bonds with ACE residues, while the peptide ARI formed eleven hydrogen bonds. The values of the hydrogen bond parameters of the best pose (ARL/I) are exhibited in Table 4.

Based on ACE’s catalytic mechanism and relevant references, three main active site pockets (S1, S1′, and S2′) of ACE were identified [40,41]. The S1 pocket includes residues Ala354, Glu384 and Tyr523, while residues Gln281, His353, Lys511, His513 and Tyr520 correspond to the S2′ pocket and the S1′ pocket only contains Glu162 residue [41]. The docking results revealed that peptide ARL established hydrogen bonds with the S1 pocket (Ala354, Glu384 and Tyr523) and the S2′ pocket (Gln281, Lys511, His513 and Tyr520) of ACE. The peptide ARI established hydrogen bonds with all aforementioned active site residues of ARL; in particular, the hydrogen bond with the residue Glu162 is also present in the docking of ARI at the S1′ pocket of ACE. The result of the docking simulation supports the theory that these three active site pockets of ACE possess different affinity for different amino acids on the substrate. Most aromatic amino acids and proline show affinity for the S1 pocket as well as Ala, Val and Leu. Ile is more favorable for the S1′ active pocket. Pro and Leu in the substrate sequence are most favorable for the S2′ pocket with regard to the affinity exerted on the enzyme [42]. In addition, the zinc (II) ion is also a critical component in ACE catalysis and is partly responsible for the binding strength between ACE and their inhibitors [40,41]. After molecular docking, it was found that the amine group of peptides ARL and ARI were close to the zinc ion and coordinating with it, which may form a distorted tetrahedral geometry (Figure 6E,F). Therefore, the ACE lost its activity. These results are to some extent in concordance with previous studies based on the molecular recognition between bioactive peptides and ACE [40,41]. According to the above results, the binding of the peptide ARL/I in the active site of ACE indicated a competitive inhibition characteristic, which is in high agreement with the results of the ACE inhibition pattern obtained from the Lineweaver–Burk plot.

### 2.6. Hemolytic Activity

**Figure 7:**
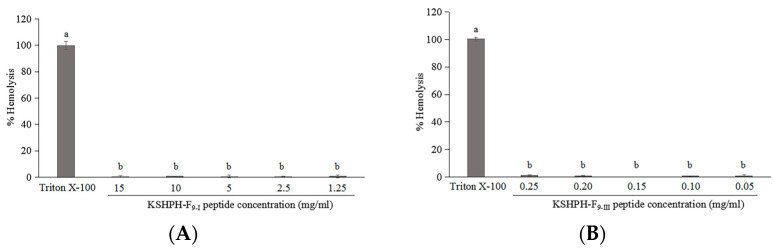
Hemolytic activity of KSHPH−F_9-I_ (**A**) and KSHPH−F_9-III_ (**B**) against rabbit red blood cells. Different superscripts note the significant differences (*p* < 0.05). All results are average ± SD from three determinations.

Hemolytic ability is among the important indexes to evaluate the safety of active peptides. Each peptide was assayed at various concentrations, the highest concentration of samples corresponding to 5 fold the highest MIC value of antibacterial peptides and the IC_50_ value of the ACE inhibitory peptide. The hemolysis percentage was below 2% for all peptides, which means no hemolytic activity was observed for all peptides (Figure 7). Generally, the bioactive peptides exhibiting no/low hemolytic activity have potential for application in the food or health-related industries [37].

## 3. Materials and Methods

### 3.1. Materials

ACE (EC 3.4.15.1) from rabbit lung, HPLC-grade acetonitrile (ACN), methanol and trifluoroacetic acid (TFA) were obtained from Sigma-Aldrich Co. LLD. (Tokyo, Japan). Hippuryl-L-histidyl-L-leucine (HHL) and papain were purchased from Wako Pure Chemical Industries, Ltd. (Osaka, Japan). All other chemicals and reagents used were of analytical grade.

Kuruma shrimp (*M. japonicus*) specimens were collected in living status from a local aquafarm in Amakusa city, Kumamoto prefecture, Japan. Shrimp heads were separated and used for hydrolysate preparation.

### 3.2. Preparation of Protein Hydrolysate of Kuruma Shrimp Heads (KSHPH)

Fresh shrimp heads were ground in an electric food processor at 4 °C. The minced shrimp heads were then homogenized in a blender with distilled water added till the final substrate concentration was 1:2 (W:V). After 5 min, the sample was adjusted to optimal pH and temperature for the activity of papain (pH 7.0; 50 °C) [43]. The hydrolysis reaction was started by the addition of enzyme (enzyme/substrate, 10,000 U/g). During hydrolysis, the pH of the mixture was maintained at the desired value by adding the 5 mol/L NaOH continuously. After hydrolysis for 4 h, the hydrolysate was heated at 95 °C for 10 min to inactivate the enzymes, followed by adjusted the pH of the hydrolysate to 7.0. Then, the protein hydrolysate was centrifuged at 12,000× *g* for 20 min at 4 °C. Next, the supernatant was recovered, freeze-dried, and then stored at 4 °C until subsequent use.

### 3.3. Determination of Molecular Weight Distribution of KSHPH

The molecular weight distribution of KSHPH was determined by high-performance size-exclusion chromatography (HPSEC) using a HPLC system (LC-20A, SHIMADZU Corporation, Kyoto, Japan) equipped with a multiwavelength detector (MD-2010 plus, JASCO Corporation, Tokyo, Japan). The Superdex 75 10/300 GL column (1 × 30 cm, GE Healthcare Biosciences, Buckinghamshire, UK) was used in this test. KSHPH was dissolved in ultrapure water, filtered through a 0.2 µm membrane filter, and then separated onto the column (elution at a flow rate of 0.4 mL/min). The HPLC system was calibrated with four molecular weight markers: Albumin (66,463 Da), Carbonic Anhydrase (30,000 Da), Cytochrome c (12,384 Da), and Aprotinin (6,512 Da).

### 3.4. Purification of Peptides

The KSHPH was fractionated using a LC-20AD HPLC system (SHIMADZU Corporation, Kyoto, Japan) of first-stage RP-HPLC on a TSKgel ODS-80TM column (250 × 4.6 mm, Tosoh Corp., Tokyo, Japan). The sample was injected at a volume of 20 µL. The column was pre-equilibrated with eluent A (water containing 0.1% TFA) for 10 min, then peptides were eluted with a linear gradient of eluent B (ACN containing 0.1% TFA) at a flow rate of 1.0 mL/min. On-line UV absorbance scans were performed at 220 nm. Major fractions were collected, lyophilized, and then their antibacterial and ACE inhibitory activities were determined. The fractions which showed strongest antibacterial and/or ACE inhibitory activities, were further separated by second-stage RP-HPLC on a Mightysil RP-18 column (150-4.6, 5 µm, Kanto Chemical Co., Inc., Tokyo, Japan). The elution was conducted at a flow rate of 0.5 mL/min using a linear gradient of ACN containing 0.1% TFA. The elution peaks were detected at a wavelength of 220 nm. After activity tests, the fractions with highest antibacterial and/or ACE inhibitory activities were collected, pooled and lyophilized, respectively. The purity of the active fractions was analyzed by using the same analytical column in the second step of RP-HPLC.

### 3.5. Antibacterial Activity

#### 3.5.1. Disc Diffusion Assay

Bacterial species tested in this study were as follows: *Staphylococcus aureus* NBRC 102135, *Micrococcus luteus* NBRC 3066, *Escherichia coli* Y1090, and *Shewanella putrefaciens* IAM 1509. The antibacterial activity of KSHPH was assessed by paper disc diffusion assay [44] on Luria–Bertani (LB) agar plates.

The strains were inoculated into 5 mL of sterile LB and incubated for 15 h at 37 °C for *S. aureus*, *E. coli* and at 35 °C for other bacterial strains. After CFU counting, the bacterial concentration was adjusted to 1.0 × 10^7^ CFU/mL with sterile saline solution. After a short vortex homogenization, 100 µL of bacteria solution was added to every plate to make it evenly coated. Filter paper discs of 8 mm diameter have been pre-sterilized by a high-pressure steam sterilizer (BS-325, TOMY SEIKO Co., Ltd., Tokyo, Japan) at 121 °C for 20 min. Using ethanol-dipped and flamed forceps, these discs were aseptically placed over nutrient agar plates seeded with the respective test microorganisms. Then, samples were added to every disc. The plates were incubated at 37 °C for 24 h for *S. aureus*, *E. coli* and at 35 °C for other bacterial strains. Antibacterial activity was measured as the diameter of the clear zone of inhibition compared to a positive control, tetracycline, and a negative control, sterile saline solution, in plates. All assays were performed in triplicate.

#### 3.5.2. Determination of Minimum Inhibitory Concentration (MIC)

MIC assays were performed using the method of Bougherra et al. [31] with some modification. A certain concentration of the peptide fraction was dissolved in distilled water and then 2-fold serial dilutions were prepared in a 96-well microplate. The test mixture of each well is comprised of 50 µL of LB medium, 50 µL of the peptide test solution and 100 µL of bacterial suspension (1 × 10^6^ CFU/mL). The inhibition of bacterial growth was monitored by measuring absorbance at 620 nm on a Multiskan FC microplate reader (Thermo Fisher Scientific Inc., Waltham, MA, USA) after incubation at 37 °C for 18 h. The absorbance of the well microplate corresponding to decreasing concentrations of the peptide fraction was compared to those of well microplates of a negative control consisting of LB medium and a positive control, tetracycline. Experiments were performed in triplicate. The MIC was defined as the lowest concentration of the peptide fraction that caused no visible increase in absorbance at 620 nm after incubation at 37 °C for 18 h without shaking.

### 3.6. ACE Inhibitory Activity

ACE inhibitory activity was measured according to the method of Cushman and Cheung [45] with some modification. This assay was performed by using HHL as substrate and UV spectrophotometry to detect the production of hippuric acid. A sample solution (80 µL) with 200 µL of HHL solution (2.5 mM) was pre-incubated at 37 °C for 5 min. ACE (20 µL, 0.2 U/mL) was added to start the reaction. After incubation at 37 °C for 60 min, the enzymatic reaction was stopped by adding 100 µL of 1 M HCl. The hippuric acid formed was extracted with 600 µL of ethyl acetate from which 400 µL was evaporated. The residue was dissolved in 1 mL of distilled water and its absorbance was measured at 228 nm. The inhibition activity was calculated using the following equation: ACE inhibition activity (%) = [1 − (S − Sb)/(C − Cb)] × 100, where C, Cb, S, and Sb represent the absorbance of the control (100% activity), the blank inhibitor (HHL alone), the sample (inhibitor peptide), and the blank sample (peptide alone), respectively. The IC_50_ value (the concentration of inhibitor being able to inhibit 50% of the ACE activity) was calculated by plotting the % ACE inhibition against the different concentrations of the peptide.

### 3.7. Peptide Identification and Structural Analysis

Peptide analysis was performed using a Waters ACQUITY UPLC system coupled to a TQ detector (Waters, Milford, MA, USA). The purified peptides (0.5 mg/mL) were dissolved in 80% ACN containing 0.1% (*v*/*v*) formic acid, and then 10 µL of the sample filtered through a 0.2 µm filter membrane was automatically injected to the UPLC system, equipped with a TSK gel Amide-80 column (3 µm, 2.0 mm ID×15 cm; TOSOH, Japan). The eluent A was MilliQ water, and the eluent B was ACN. The flow rate of elution was 0.2 mL/min and the gradient consisted of 90% B for 5 min, followed by a linear decrease to 50% B in 20 min. The UPLC eluent was directly injected into the TQ detector, which was equipped with an electrospray ionization (ESI) source.

MS spectra were recorded in the positive mode using the full-scan method firstly from 100 to 2000 *m*/*z*. The molecular mass of peptides was detected by a charged [M + H]^+^ state analysis in the mass spectrum and the amino acid sequence was identified by tandem MS analysis. The amino acid sequence of peptides was performed using MassLynx software version 4.1 and confirmed by manual validation. The optimum 3D structure of identified peptides was modeled by the Hyperchem 8.0 software (Hypercube, Gainesville, FL, USA). Several physicochemical properties of peptides were calculated using a peptide property calculator (http://www.novoprolabs.com/tools/calc_peptide_property, accessed on 24 October 2022). The structural characteristics of the peptide were deduced by a mathematical model of the Network Protein Sequence analysis Internet server of the Pole Bio-informatique Lyonnais (http://pbil.ibcp.fr, accessed on 27 October 2022).

### 3.8. Determination of ACE Inhibition Pattern

To clarify the ACE inhibition pattern of the purified peptide, the assay was conducted with various concentrations of HHL substrate (4, 2, 1, and 0.5 mM) in the absence and presence of different concentrations of the peptide inhibitor (40 and 80 µg/mL) according to the method of Wu et al. [38]. Lineweaver–Burk plots of 1/absorbance versus 1/HHL were used to determine the type of enzyme inhibition. Additionally, the inhibition constant Ki of the ACE inhibitory peptide was investigated by a Dixon plot.

### 3.9. The Molecular Docking Simulation

To elucidate the inhibitory mechanism of the peptide to ACE, the molecular docking of the identified peptides with ACE was studied. The crystal structure of ACE used in this research was downloaded from the RCSB PDB Protein Data Bank (http://www.rcsb.org, accessed on 31 October 2022) with the code 1O86.pdb (ACE-lisinopril complex), which represents the human tACE in complex with lisinopril at 2 Å resolution. Molecular docking was conducted using the flexible docking tool of AutoDock 4.2 (TSRI, La Jolla, CA, USA). Before docking, all water molecules and the inhibitor lisinopril were removed, whereas the zinc and chloride atoms were retained in the active site. Then, the polar hydrogens and atom type were added to the ACE model. The docking runs were carried out with a radius of 4 Å, with coordinates x: 40.484, y: 33.632, and z: 47.188. The Lamarckian genetic algorithm (LGA) was used to search the optimal binding sites during the docking simulation. Lisinopril was docked as a reference for active sites. The best ranked docking pose of the purified peptide in the active site of ACE was obtained according to the scores and binding energy values.

### 3.10. Hemolytic Activity Assay

The hemolytic activity of the purified peptide fractions was determined as described by Khueychai et al. [37] with some modifications. A volume of 5 mL of rabbit red blood cells was centrifuged for 5 min at 2000× *g* to isolate the erythrocytes, washed three times in an isotonic phosphate-buffered saline (PBS) solution (pH 7.4) until the buffer turned clear and resuspended in PBS to the initial blood volume. The cell suspensions of 50 µL (final 2% erythrocytes) were mixed with various concentrations of the peptide fraction of 50 µL, and then added to PBS solution to give a final volume of 200 µL. The mixtures were then incubated at 37 °C for 30 min. After incubation, the mixtures were centrifuged at 2000× *g* for 5 min, and the supernatant was transferred to a new 96-well plate. Release of hemoglobin was monitored by measurement of absorbance at 540 nm. PBS solution and 1% (*v*/*v*) Triton X-100 were used as the negative and positive controls, respectively. The percentage of hemolysis was calculated as follows: hemolysis % = (At − Anc)/(Apc − Anc) × 100, where At, Anc, and Apc represent the absorbance of the test, the absorbance of the negative control, and the absorbance of the positive control, respectively.

### 3.11. Statistical Analysis

All tests were conducted at least in triplicate. Statistical analyses were performed using SPSS version 25.0 software, and the significant differences were determined with a 95% confidence interval (*p* < 0.05) using Duncan’s multiple range test.

## 4. Conclusions

Conclusively, instead of low-value utilization, such as aquatic feed processing, the head wastes of kuruma shrimp are a desirable ingredient to prepare bioactive peptides. In this study, the antibacterial peptide (VTVP) and the ACE inhibitory peptide (ARL/I) from KSHPH have been successfully purified using two stages of RP-HPLC and then identified by LC–MS/MS. The peptide VTVP exhibited better antibacterial potency against the tested Gram-positive strains (*S. aureus* and *M. luteus*), with MIC values of 1.62 and 2.00 mM, respectively. The peptide ARL/I showed potent ACE inhibitory activity and inhibited ACE in a competitive manner. Molecular docking revealed that the hydrogen bonds were mainly interaction forces responsible for binding between ARL and ACE active sites (S1 and S2′ pockets), and between ARI and ACE active sites (S1, S1′, and S2′ pockets). Moreover, the peptide ARL/I also form a coordination bond with a Zn^2+^ site that may cause ACE deactivation. Remarkably, the purified peptides have no hemolytic activity in rabbit red blood, even when tested at high concentrations. Therefore, it is anticipated that the new finding in this research could provide promising opportunity for developing potential supplementary and therapeutic agents for the food and pharmaceutical industries.

## Figures and Tables

**Table 1:** Antibacterial activity of KSHPH.

Bacterial Strains	Zone of Inhibition (mm)		
	A	B	C
*S. aureus* NBRC 102135	+	++	+++
*M. luteus* NBRC 3066	–	+	++
*E. coli* Y1090	–	–	–
*S. putrefaciens* IAM 1509	–	–	–

KSHPH (dry weight): A, 20 mg; B, 30 mg; C, 40 mg. Inhibition zones: +++, 20~16 m; ++, 15~11 mm; +, <11 mm; and –, no activity.

**Table 2:** Purification of the ACE inhibitory peptide of shrimp head hydrolysate from *M. japonicus*.

Purification Step	IC_50_ Value (mg/mL) ^a,b^	Purification Fold
KSHPH	1.90 ± 0.03	1.00
KSHPH-F_9_	0.97 ± 0.04	1.96
KSHPH-F_9-III_	0.045 ± 0.005	42.22

^a^ IC_50_ values was defined as the concentration of inhibitor required to inhibit 50% of ACE activity. ^b^ Values are presented as the mean ± SD (*n* = 3).

**Table 3:** MIC values of antibacterial peptides against bacteria strains.

	Bacterial Strains	MIC Values (mg/mL)					
		KSHPH−F_9_		KSHPH−F_9-I_		Tetracycline	
		mg/mL	mM	Mg/mL	mM	mg/mL	mM
Gram (+)	*S. aureus* NBRC 102135	5.00 ^a^	NA	0.67 ^a^	1.62 ^a^	0.0017 ^a^	0.0038 ^a^
	*M. luteus* NBRC 3066	5.00 ^a^	NA	0.83 ^a^	2.00 ^a^	0.0017 ^a^	0.0038 ^a^
Gram (−)	*E. coli* Y1090	16.67 ^b^	NA	3.33 ^b^	8.03 ^b^	0.3333 ^b^	0.7499 ^b^
	*S. putrefaciens* IAM 1509	6.67 ^a^	NA	1.00 ^a^	2.41 ^a^	0.0025 ^a^	0.0056 ^a^

NA indicates not applicable, since the sample contains multiple peptides. Different superscript letters within a column mark significantly different values (*p* < 0.05).

**Table 4:** Hydrogen bonds observed between best bioactive peptide poses obtained from docking results and ACE.

	Hydrogen Bond Obtained from Molecular Docking			
	ARL	Distance (Å)	ARI	Distance (Å)
Glu162: O(E2)	×		√	2.17
Gln281: N(E2)	√	2.21	√	1.93
Ala354: O	√	2.13	√	2.22
Ala354: O			√	2.59
His383: N(E2)	√	3.35	√	3.35
Glu384: O(E2)	√	1.78	√	1.90
Glu384: O(E2)	√	2.08	√	2.27
Glu384: O(E2)	√	2.18		
Lys511: N(Z)	√	1.69	√	1.70
His513: N(E2)	√	2.20	√	2.22
Tyr520: O(H)	√	2.14	√	1.92
Tyr523: O(H)	√	1.81	√	1.71

In XXX123: Y(00), XXX represents the abbreviation of amino acid molecule, 123 represents the serial number of this amino acid molecule on ACE, Y represents the atom involved into the interaction and 00 represents the serial number of this atom on this amino acid molecule. √, existence of hydrogen bond interactions; ×, non-existence of hydrogen bond interactions.

## Data Availability

Not applicable.
